# Antidepressants Stimulate Hippocampal Neurogenesis by Inhibiting p21 Expression in the Subgranular Zone of the Hipppocampus

**DOI:** 10.1371/journal.pone.0027290

**Published:** 2011-11-04

**Authors:** Robert N. Pechnick, Svetlana Zonis, Kolja Wawrowsky, Rosemarie Cosgayon, Catherine Farrokhi, Liliana Lacayo, Vera Chesnokova

**Affiliations:** 1 Department of Psychiatry and Behavioral Neurosciences, Cedars-Sinai Medical Center, Los Angeles, California, United States of America; 2 Division of Endocrinology, Department of Medicine, Cedars-Sinai Medical Center, Los Angeles, California, United States of America; 3 Brain Research Institute, University of California Los Angeles, Los Angeles, California, United States of America; Rikagaku Kenkyūsho Brain Science Institute, Japan

## Abstract

The relationships among hippocampal neurogenesis, depression and the mechanism of action of antidepressant drugs have generated a considerable amount of controversy. The cyclin-dependent kinase (Cdk) inhibitor p21^Cip1^ (p21) plays a crucial role in restraining cellular proliferation and maintaining cellular quiescence. Using *in vivo* and *in vitro* approaches the present study shows that p21 is expressed in the subgranular zone of the dentate gyrus of the hippocampus in early neuronal progenitors and in immature neurons, but not in mature neurons or astroglia. *In vitro*, proliferation is higher in neuronal progenitor cells derived from p21-/- mice compared to cells derived from wild-type mice. Proliferation is increased in neuronal progenitor cells after suppression of p21 using lentivirus expressing short hairpin RNA against p21. *In vivo,* chronic treatment with the non-selective antidepressant imipramine as well as the norepinephrine-selective reuptake inhibitor desipramine or the serotonin-selective reuptake inhibitor fluoxetine all decrease p21 expression, and this was associated with increased neurogenesis. Chronic antidepressant treatment did not affect the expression of other Cdk inhibitors. Untreated p21-/- mice exhibit a higher degree of baseline neurogenesis and decreased immobility in the forced swim test. Although chronic imipramine treatment increased neurogenesis and reduced immobility in the forced swim test in wild-type mice, it reduced neurogenesis and increased immobility in p21-/- mice. These results demonstrate the unique role of p21 in the control of neurogenesis, and support the hypothesis that different classes of reuptake inhibitor-type antidepressant drugs all stimulate hippocampal neurogenesis by inhibiting p21 expression.

## Introduction

The subgranular zone (SGZ) of the dentate gyrus of the hippocampus and the subventricular zone, which lines the border between the striatum and the lateral ventricle, are known to produce new neurons throughout life. Neuronal stem cells express glial fibrillary acidic protein (GFAP) [1]. Unlike in the subventricular zone, in the hippocampus most of the cells isolated by stem cell neurosphere assays express SOX2 and nestin, both markers of quiescent neuronal stem cells and amplifying neuronal progenitors [2]; however, very few cells express GFAP. Therefore, hippocampal neural stem cells are referred as “neuronal progenitor cells” (NPC) [2], [3].

The relationships among hippocampal neurogenesis, depression and the mechanism of action of antidepressants have generated a considerable amount of interest and controversy. Most antidepressant drugs produce a rapid increase in synaptic levels of norepinephrine and/or serotonin; however, the onset of clinical improvement usually takes 3–4 weeks [4]. Thus, the initial increase in levels of these biogenic amines must trigger downstream events that after some time lead to a therapeutic effect. It has been suggested that stimulation of hippocampal neurogenesis is one of these downstream events. Maturation of newly developed neurons also requires about 3–4 weeks, and different classes of antidepressants stimulate the proliferation of neuronal progenitors [5], [6], [7] and increase the survival of newly developed neurons [8]. In addition, ablation of neurogenesis by irradiation reduces some of the effects antidepressants [9], [10], [11]. Thus, intact hippocampal neurogenesis is required for at least some of the behavioral effects of antidepressants in animal models. These findings point to a potential mechanistic link between neurogenesis and the mechanism of action of antidepressant drugs.

The end-point molecular mechanisms regulating hippocampal neurogenesis are not clear. In mammalian cells, the control of proliferation primarily occurs in the G1 phase of the cell cycle [12]. Cyclin/Cdk complexes are negatively regulated by two families of Cdk inhibitors: the Ink4/Arf family (p15, p16, p18 and p19); and the Cip/Kip family (p21, p27 and p57) [13]. By inhibiting cyclin/Cdk activity, Cdk inhibitors stop the transition from the G1 to the S phase. The Cdk inhibitor p21^Cip1^ (p21) plays a crucial role in restraining proliferation and maintaining cellular quiescence [14].

Previously, we showed that p21 is expressed in the SGZ of the hippocampus [15]. In the present study we examined in details the specific cell types that express p21, and the functional significance of its expression in the SGZ are defined. We analyzed the effects of different classes of antidepressants on SGZ p21 expression and examined neurogenesis in p21-/- mice at baseline and after chronic imipramine treatment. Behavior of wild-type (WT) and p21-/- mice after chronic imipramine treatment was compared and contrasted using the forced swim test. The results show that p21 is expressed in transit-amplifying progenitors and neuroblasts and negatively regulates proliferation of these cells. Chronic treatment with different classes of antidepressant drugs all inhibit the expression of p21, but do not affect the expression of other Cdk inhibitors, and this is associated with increased neurogenesis. These results suggest that p21 uniquely regulates NPC proliferation, and by inhibiting p21, reuptake inhibitor-type antidepressants release proliferation restraint and increase neurogenesis in the hippocampus.

## Methods

### Experimental animals

This study was carried out in strict accordance with the recommendations in the Guide for the Care and Use of Laboratory Animals of the National Institutes of Health. The protocol was approved by the Institutional Animal Care and Use Committee at Cedars-Sinai Medical Center (project# 2263). C57Bl/6 and p21-/- (Cdk1a^tm1Tyj^) mice were originally purchased from the Jackson Laboratory, but currently are bred in our laboratory. p21-/- mice were backcrossed to the C57Bl/6 genetic background 6 times prior to testing. p21+/- females and males were used for breeding, and both WT and p21-/- animals were obtained from the same litters. Two month old male mice were used for the experiments. For Western blot analyses and for obtaining NPC, the mice were sacrificed by cervical dislocation, the brains removed and rapidly cooled in ice-cold saline and the hippocampi were dissected out [16]. For the immunohistochemistry studies the mice were anesthetized with isoflurane and perfused with paraformaldehyde (4%). Separate groups of mice were used for the behavioral studies.

### Drugs

The doses were based upon those that produce behavioral changes (reduce immobility in the forced swim test), increase neurogenesis and/or produce neurochemical changes reflective of antidepressant action [17], [18], [19]. Fluoxetine hydrochloride (Spectrum Chemicals) (10 mg/kg/day) initially was dissolved in distilled water and diluted in normal saline to volume prior to injection. Imipramine hydrochloride (Sigma-Aldrich) (10 mg/kg/day) and desipramine hydrochloride (Sigma-Aldrich) (20 mg/kg/day) were dissolved in normal saline. The doses are expressed as the salt form. Drugs were injected i.p. in a volume of 1 ml/100 g body weight once a day for 3 weeks.

### Bromodeoxyuridine (BrdU) incorporation

BrdU incorporation was assessed as described previously [15]. The mice were injected every two hr with BrdU (Sigma-Aldridge, 100 mg/kg/i.p) for a total of three injections and then sacrificed 24 hr after the first BrdU injection. The entire left half of the brain was cut into sagittal sections (5 µm) and processed using a BrdU Labeling and Detection Kit (Roche Applied Biosystems). Sections were coded for blind data analysis. Cutting of sections from 0.36 to 0.6 mm lateral to the midline was carried out (55). Every third section (total 30 sections) was counted under a x100 objective and the sum was multiplied by 3 to estimate the total number of BrdU-positive cells in the region. Cells were counted if they were in or touching the SGZ and excluded if they were more than two cell diameters from the GCL [7]. Some sections were double-labeled to detect DCX, and BrdU-positive cells were examined by confocal microscopy to determine co-localization with DCX.

### Protein isolation and Western blot analysis

Analyses were conducted as described previously [20]. The following antibodies were used: p21, p18, p27 β-actin (Santa Cruz Biotechnology) and cleaved caspase 3 (Abcam). DCX and NeuN antibodies were the same as used for the immunohistochemistry studies (see below).

### Adult NPC cultures

Cultures were prepared and conducted according to published protocols [1], [2], [21]. Two month old WT and p21-/- mice were sacrificed, the hippocampi dissected and dissociated using Papain Dissociation System (Worthington Biochemicals). The NPC cells were isolated and cultured using Neural Stem Cell Expansion Kit Neurosphere System in serum-free neurobasal A-medium (R&D Systems). The single-cell suspension was re-suspended in DMEMF-12 medium supplemented with N-2 Plus Media Supplement, 2 mM L-glutamine, 100 U/ml penicillin, 100 µg/ml streptomycin, 10 ng/ml FGF-2 and 20 ng/ml EGF. Under these conditions only stem/neuronal progenitors survive and form spheres. Cells were dispersed and passaged weekly and passages 2 -4 were used for the experiments. Differentiation was induced by growing in Complete NeuroCult NSC Differentiation Medium (StemCell Technologies) in the absence of FGF-2 and EGF, and 5×10^4^ cells/ml were plated on ECL cell Attachment Matrix coated coverslips (Upstate, 5–10 µg/cm^2^) in 24 well plates and cultured for 3 days (to detect DCX-positive cells) or for 8 days. Cells were then fixed in 4% paraformaldehyde and immunocytochemistry performed to detect neuronal markers.

### Sphere self-renewal analysis

NPC cells were isolated from 2 month old WT and p21-/- mice. After the culture was established, at passage 2 the spheres were collected, dispersed and 0.5 cells/well were seeded in 96 well plates in quadruplicate. Ten days later the number of spheres/well was counted as a percentage of cells capable of forming spheres. All spheres were then collected, dispersed and the total number of cells derived from the spheres was counted in each culture.

### Transient transfection of NPC with short hairpin RNA (shRNA) against p21

Lentiviral-based particles permit efficient infection and integration of specific shRNA constructs into non-dividing cells such as neuronal precursors. Mission®Lentiviral Transduction Particles (Sigma-Aldrich) consist of sequence-verified p21 shRNA cloned into pLKO.1-puro lentiviral vectors. The viral titer was greater than 1.5×10^7^ transducing units. At passage 2 or 3 the spheres were collected, dispersed and plated at 5000 cell/well in 24-well plates in duplicate. Transducing lentiviral particles expressing p21 shRNA or control non-targeting shRNA were added in the amount calculated to reach optimal multiplicity of infection (MOI) of 20 units. After 48 hr the cells were re-infected with the same amount of lentiviral particles. Cells were fixed in 4% paraformaldehyde 48 hr after the second infection.

### Immunohisto- and cytochemistry studies

Brain paraffin sections (5 µM) or coverslips were double-labeled with primary antibodies conjugated with Alexa 488 and Alexa 568 fluorescent dyes (Molecular Probe)[20]. The following antibodies were used: p21 (BD Pharmingen), NeuN (Chemicon), SOX2 and GFAP (both Millipore), BrdU (Santa Cruz Biotechnology), DCX, nestin, Ki67 (all Abcam) and Tuj-1 (Stem Cell Technology). DNA (nuclei) was stained with DAPI (Prolong Gold, Invitrogen). Three independently performed stains were analyzed for each antibody. Immunoreactive cells were determined in 3–10 random fields (total number of cells between 500 and 5,000 depending on experiment). Multiparameter fluorescent microscopy and Leica Confocal Software was used to identify localization and co-localization of these proteins.

### Forced Swim Test (FST)

The FST is used primarily as a screen for detecting antidepressant activity, but it also has been used to study animal models of depression [22]. Immobility (defined as the absence of active, escape-oriented behaviors such as swimming, jumping, rearing, sniffing or diving) has been associated with depression-like behavior (i.e., behavioral despair or learned helplessness). The forced swim test was performed as described previously [23], [24]. The level of illumination in the rooms during experimental testing was 325 lux. The mice were individually placed into a Plexiglas cylinder (25 cm height, 10 cm diameter) containing 16 cm of water maintained at 22–24°C. The duration of the test was 6 min, and the behavioral responses were videotaped. At a later date the videotapes were viewed, and trained raters recorded the total duration of time spent immobile (defined as the absence of active, escape-oriented behaviors such as swimming, jumping, rearing, sniffing or diving). The raters were blind to the experimental treatment.

### Statistical analysis

Independent-samples t-tests were used to analyze paired and planned comparisons. Groups with more than 2 comparisons were analyzed using one-way ANOVA (to determine the effects of treatments) followed by the Dunnett's test for pairwise comparisons to controls, or two-way ANOVA (to determine the effects of genotype and treatments) followed by the Newman-Keuls posthoc test for all pairwise comparisons. In one instance where homogeneity of variance was not met, the non-parametric Kruskal-Wallis test was used followed by a multiple comparisons of means to detect differences between groups. The level of significance for all analyses was set at p<0.05.

## Results

### p21 is expressed in hippocampal neuronal progenitor cells *in vivo*

**Figure 1:**
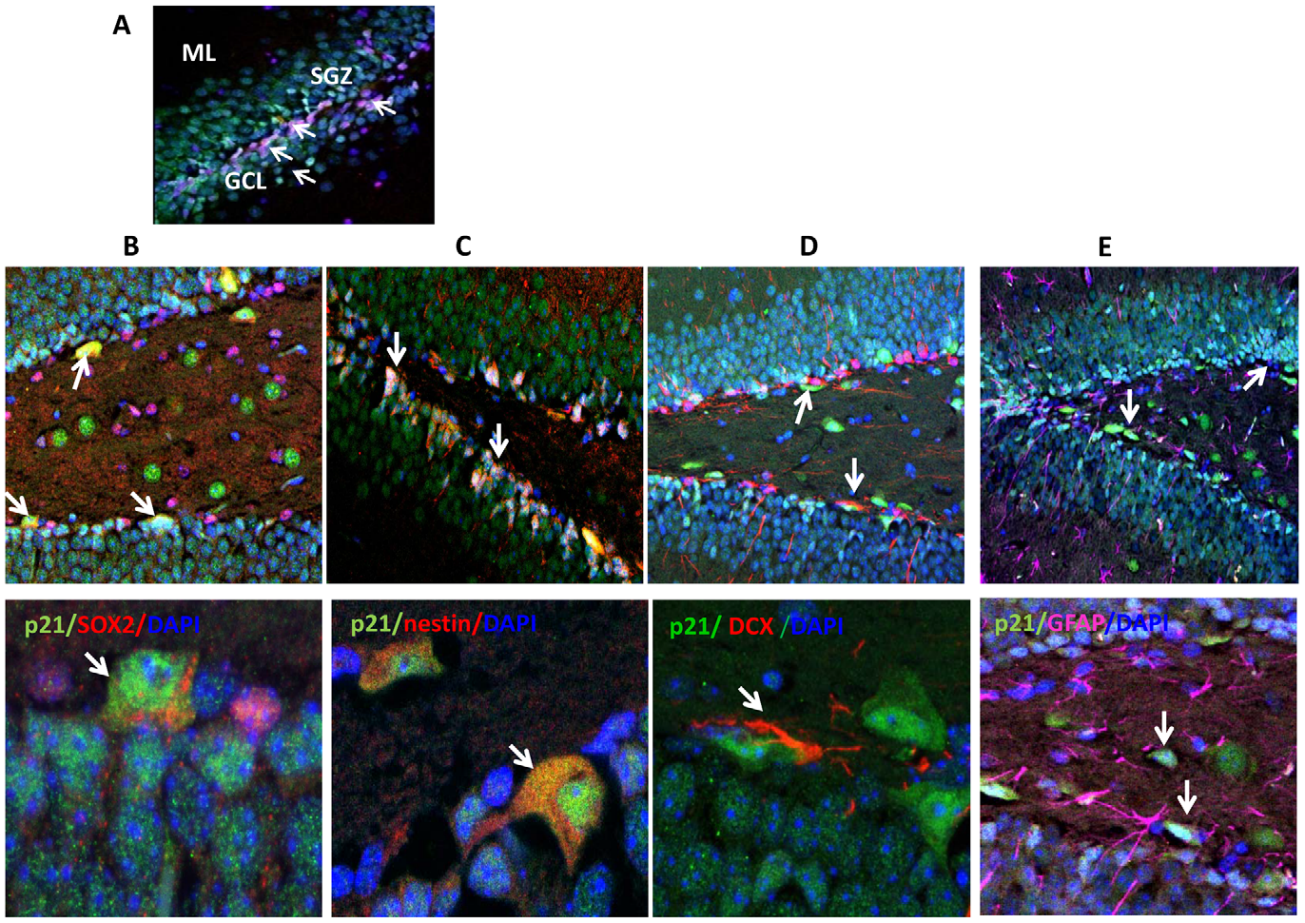
p21 is expressed in neuronal progenitors in the SGZ of the hippocampus. **A**) The confocal image shows that p21 (red) is expressed in the SGZ of the dentate gyrus and is not expressed in mature neurons (NeuN, green) in the GCL. Here, and in other confocal images, nuclei are stained with the DNA specific dye DAPI (blue). p21-positive cells appear pink. p21 is expressed mostly in nuclei; however, some cytoplasmic staining also is observed. SGZ, subgranular zone; GCL, granular cell layer; ML, molecular layer; **B–E**) Confocal images depicting p21 co-localization with neuronal markers in the SGZ of the hippocampus. p21-positive nuclei line the SGZ and appear light blue. Upper panel-low magnification (x20), lower panel–higher magnification (x63). **B**) p21 (green) is co-localized with SOX2 (intranuclear, red). Cells that express both proteins appear yellow; cells that expressed SOX2 only appear pink; **C**) p21 (green) is co-localized with nestin (cytoplasmic, red); **D**) p21 (green) is co-localized with DCX (cytoplasmic, red); **E**) p21 (green) is not co-localized with GFAP (cytoplasmic, pink). Some of p21 positive cells are marked with arrows.

In the hippocampus, NPC constitute slow dividing quiescent radial “stem” cells (GFAP^+^/SOX2^+^ or GFAP^+^/nestin^+^ type 1 cells), transit amplifying progenitors (GFAP^−^/SOX2^+^ and GFAP^−^/nestin^+^ type 2a cells, or GFAP^−^/nestin^+^/DCX^+^ type 2b cells), and neuroblasts (DCX^+^/βIII-tubulin^+^), but not mature neurons (NeuN^+^; type 3 cells) [2], [25]. Previously, it was shown that in the hippocampus, p21 is expressed exclusively in the SGZ, in the neurogenic niche [15]. Using specific neuronal markers, immunofluorescent confocal analysis confirmed this previous observation and showed that p21 is not expressed in the granule cell layer (GCL) where mature neurons reside (Fig. 1A). Three brain sections per animal derived from 3 mice were analyzed for GFAP-, SOX2-, nestin- and p21 co-localization. p21 expression was not detected in GFAP^+^/SOX2^+^ or in GFAP^+^/nestin^+^ cells (data not shown), indicating that p21 is not expressed in radial neuronal stem cells. In the SGZ, p21 is co-localized with SOX2 and with nestin in amplifying neuronal progenitors (Fig. 1B,C). Whereas 26±3.7% of SOX2^+^ cells also were positive for p21, more than 50% (56±10.4%) of nestin^+^ cells expressed p21. p21 is expressed in DCX^+^ neuroblasts (Fig. 1D), but not in differentiated GFAP^+^ astroglia (Fig. 1E). Thus, p21 is expressed in transient amplifying neuronal progenitors and early developing neurons.

### p21 is expressed in hippocampal-derived NPC *in vitro*

**Figure 2:**
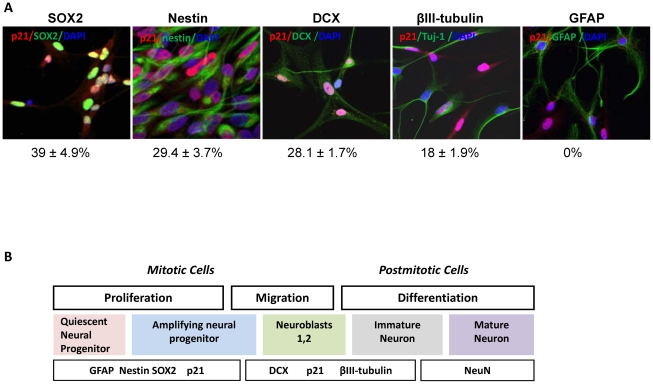
p21 is expressed in hippocampal NPC of a neuronal lineage. Cultured neuronal progenitors were stained for p21 (intranucelar, red) and various neuronal markers. **A**) co-localization of p21 and SOX2 (green, intranuclear, double-positive nuclei appear yellow; **B**) co-localization of p21 and nestin (green, cytoplasmic); In differentiating NPC, p21 co-localizes with **C**) DCX (green, cytoplasmic); and with **D**) Tuj-1 (green, cytoplasmic), but not with **E**) GFAP (green, cytoplasmic). Numbers indicate the percentage of p21-positive cells among the total number of cells expressing a respective marker. Counting was performed in triplicate (>500 cells per sample). Data are presented as mean ± SEM.

Hippocampal-derived NPC were isolated using the neurosphere assay. Sphere-forming floating neural progenitors were cultured in serum-free neurobasal A-medium under proliferating conditions (see Methods) for 7 days, then the spheres were collected and dispersed to individual cells, plated on polyornithine-covered culture dishes and after 3 passages they were analyzed. We did not find p21 expression in GFAP^+^/SOX2^+^ or in GFAP^+^/nestin^+^ cells, therefore double-staining for p21- and nestin or SOX2 was carried out. In three separate experiments, more than 3000 cells were analyzed. The results show that 39±4.9% of SOX2^+^ cells and 29.4±3.7% of nestin^+^ cells (Fig. 2A) also express p21. Therefore, *in vitro*, p21 is expressed by transient amplifying neuronal progenitors prior to neuronal differentiation. Next, it was determined whether p21 is expressed in early stage developing neurons. NPC were allowed to differentiate for only 3 days, at which time a proportion of the cells start to express the markers of neuroblasts and became DCX^+^. In three separate experiments, 1600 DCX^+^ cells were analyzed, and 28.1±1.7% expressed p21 (Fig. 2A), showing that p21 is expressed in neuroblasts. Unfortunately, there is no good marker for early stage cells committed to glial lineage, so it is difficult to assess whether p21 is also expressed in early glial progenitors. After NPC were allowed to differentiate for 8 days they lose nestin and SOX2, and generate primarily young neurons and astroglia [26], [27], [28]. Cells were double-stained for p21 and GFAP, a marker for differentiated astroglia in these conditions, and for p21 and βIII-tubulin, a marker of immature post-mitotic neurons that is recognized by antibodies to Tuj-1. The number of cells co-expressing GFAP and p21, and Tuj-1 and p21 were counted in three separate experiments and a total 3000 cells were counted for each antibody. p21 was expressed in 18±1.9% of Tuj-1^+^ cells (Fig. 2A) . Confirming the *in vivo* observations, p21 was not detected in cells that differentiated into GFAP^+^ cells (astroglia) (Fig. 2A). Thus, p21 is expressed in progenitors committed to a neuronal lineage, and its expression is negatively correlated with the degree of differentiation (Fig. 2B).

### p21 deletion increases NPC proliferation

**Figure 3:**
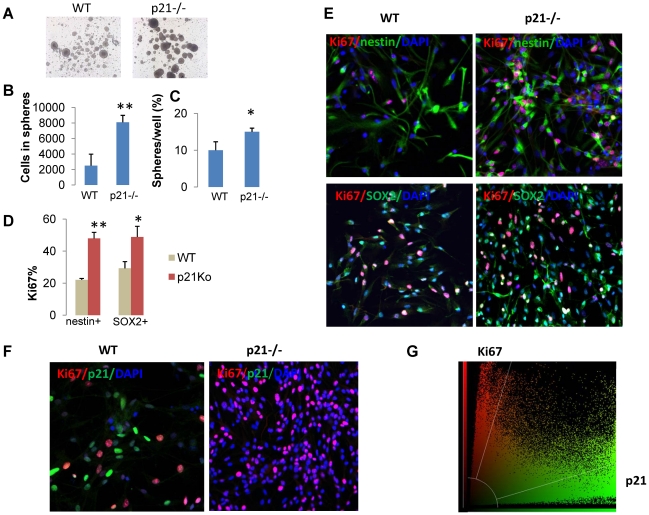
Increased proliferation of NPC derived from p21-/- mice. **A**) The spheres derived from WT and p21-/- hippocampi 7 days after plating. Note bigger size and more cellularity in p21-/- spheres; **B**) Seven days after seeding, the NPC spheres were collected, dispersed and the number of cells was counted; **C**) Sphere self-renewal analysis. NPC spheres were dispersed and cells were seeded at 0.5 cell/well into 96-well plates. The percent of cells forming new spheres was determined after 10 days; **D**) The percent of Ki67-positive cells co-expressing nestin or SOX2. Counting was performed in triplicate (>500 cells per sample). Data are presented as a mean ± SEM, *, p<0.05, **, p<0.01; **E**) Representative confocal images of proliferating NPC derived from WT and p21-/- hippocampi and stained for Ki67 (intranucelar, red) and nestin (cytoplasmic, green) or SOX2 (intranuclear, green). p21-/- NPC exhibit more Ki67-positive cells; **F**) Proliferating neuronal precursors derived from WT and p21-/- hippocampi and stained for Ki67 (intranucelar, red) and p21 (intranuclear, green). Note that WT NPC cells positive for Ki67 are negative for p21; **G**) Double-labeling analysis of WT NPC with a color-coded fluorogram. The confocal image shows the absence of co-localization between p21 (green) and Ki67 (red).

Sphere formation, proliferation and differentiation of NPC derived from the hippocampi of WT and p21-/- mice were compared. NPC were isolated and cultured in growth medium. Seven days after plating, the size of the spheres derived from the hippocampi of p21-/- mice was markedly larger than those derived from WT mice (Fig. 3A). All floating spheres were then collected, dispersed and the number of cells was counted. The total number of cells in the spheres derived from p21-/-mice was significantly (4-fold) higher than that from WT mice (Fig. 3B). Sphere self-renewal analysis was performed to analyze the potential to form spheres. The percentage of cells able to form new spheres was higher in the cell culture derived from p21-/- mice compared to those derived from WT mice (15±1 vs 10±2.3 sphere per well respectively, p<0.05) (Fig. 3C). As an additional measure of proliferation potential, NPC were grown on coated slides for 1 day and then fixed and stained for Ki67 and nestin, or SOX2. The number of nestin^+^ and SOX2^+^ cells co-expressing Ki67 was higher in NPC derived from p21-/- mice compared to WT mice (Fig. 3D and 3E). Thus, NPC derived from hipocampi of p21-/- mice have increased proliferation and self-renewal potentials. To test whether cells expressing p21 proliferate, WT and p21-/- NPC were fixed and double stained for p21 and Ki67. In WT cell culture, cells positive for p21 were negative for Ki67, indicating that cells that express p21 do not proliferate (Fig. 3F). Double–labeling confocal analysis of WT NPC using a color-coded fluorogram confirmed that these two proteins rarely co-express (Fig. 3G). These results indicate that when p21 is absent, proliferation is increased, whereas when p21 is present, proliferation is restrained.

### Transient suppression of p21 increases NPC proliferation

**Figure 4:**
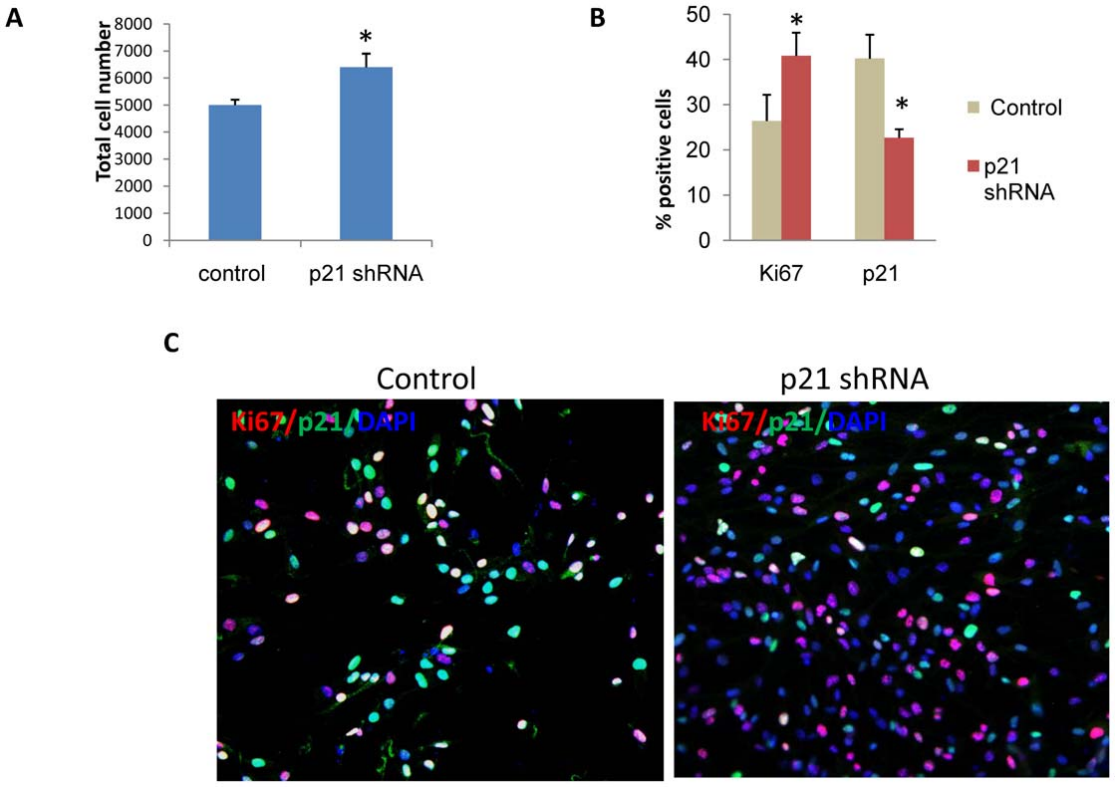
Conditional suppression of p21 enhances proliferation of WT NPC. **A**) One thousand WT NPC cells were kept in proliferating conditions for 4 days. Cells were cultured in 24-well plates in triplicates. The mean number of cells at the time of collection; **B**) Cells were fixed and stained for Ki67 or p21 . The graph depicts the percentage of p21- or Ki67-positive cells in each culture. Counting was performed in triplicates (>500 cells per sample). Data are presented as a mean ± SEM, *, p<0.05. Two independent experiments were conducted, and representative results are shown; **C**) Representative confocal images of NPC treated with either control lentivirus (control) or lentivirus expressing p21 shRNA. The cells were stained for Ki67 (intranucelar, red) and p21 (intranuclear, green). Note the increased number of Ki67-positive and the decreased number of p21-positive cells after p21 shRNa treatment.

To account for the possibility of compensatory alterations in p21-/- mice during development, conditional loss of function studies were carried out using lentivirus expressing shRNA against murine p21. Proliferating NPC cultures were infected with lentivirus expressing shRNA or lentivirus expressing non-targeting shRNA (control). The cells were collected, fixed and double-stained for p21 and Ki67. Although the same number of cells was initially plated (1,000 cells), at the time of collection there were significantly more cells in NPC cultures treated with p21 shRNA than in the control cultures (6,400±508 vs 5,000±202 per cover slip, respectively; p<0.05, Fig. 4A). In NPC treated with shRNA targeted against p21, the percentage of Ki67^+^ proliferating neuronal precursors was increased significantly (from 26.4 ±5.8% t0 40.2±5.1%, respectively, p<0.05), and at the same time the percentage of p21^+^ cells was decreased (40.83±5.3% to 22.67±1.9%, respectively, p<0.05 for both) (Fig. 4B and C), confirming that when p21 is suppressed the proliferation of neuronal progenitors is increased.

### p21 is down-regulated and neurogenesis is increased by chronic antidepressant treatment in WT mice

**Figure 5:**
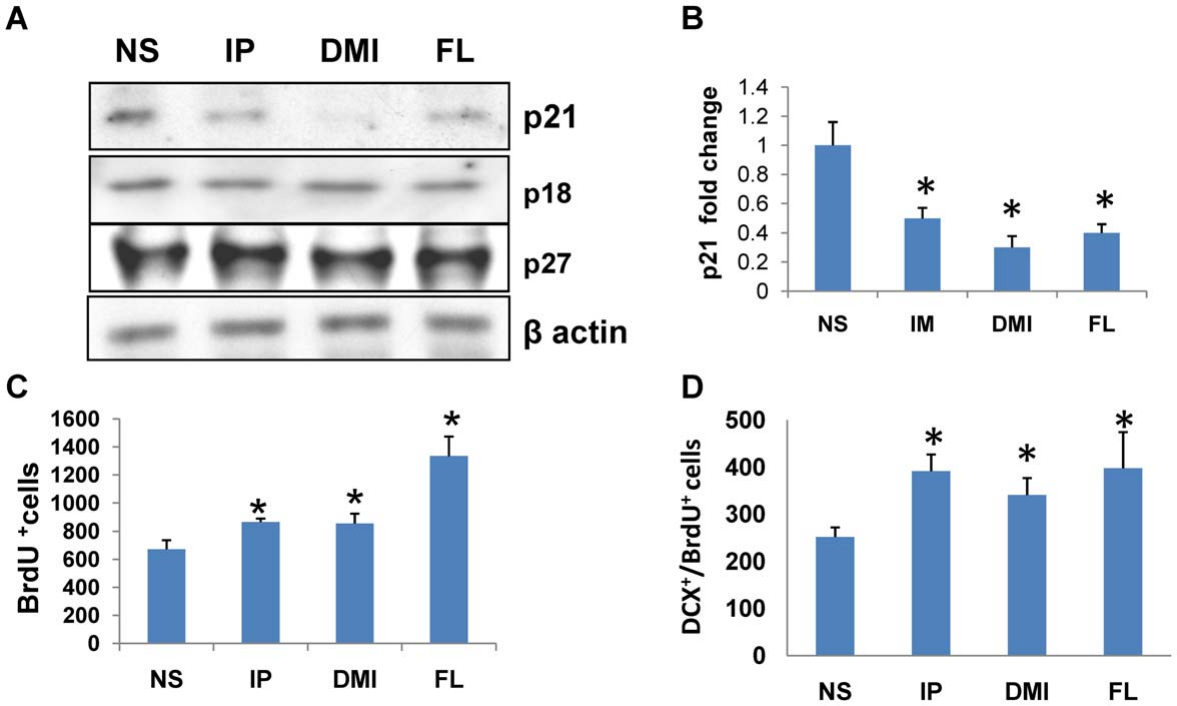
Chronic treatment with antidepressants suppressed p21 expression and increased neuronal proliferation in the SGZ of hippocampus. **A**) Western blot analysis of p21 and other Cdk inhibitors in hippocampus of mice chronically treated with normal saline (NS), imipramine (IP), desipramine (DMI) or fluoxetine (FL). For each sample hippocampi from 5 mice/group were pooled; **B**) Quantitative analysis of three independent experiments. The intensity of p21 bands was measured, normalized to the loading control (β actin) and the ratios were corrected to NS control to quantify relative changes; **C**) Number of BrdU^+^ cells. Non-parametric Kruskal-Wallis test [(H3,N = 20) = 11.75, p<0.008]. **D**) Number of BrdU^+^/DCX^+^ cells. One way ANOVA followed by Dunnett's post-hoc test [(F3,16) = 3.88, p = 0.029 for all groups] in the SGZ. For each sample hippocampi from 5 mice/group were analyzed. Data are presented as a mean ± SEM.

Previously we showed that chronic treatment with the nonselective tricyclic antidepressant imipramine markedly decreased hippocampal p21 mRNA and protein levels [15]. In order to determine whether other classes of reuptake inhibitor-type antidepressants have similar effects on hippocampal p21 expression, WT mice were injected daily for 3 weeks with saline or one of the following antidepressant drugs: the nonselective antidepressant imipramine (10 mg/kg/i.p.); the selective norepinephrine reuptake inhibitor desipramine (20 mg/kg/i.p.); or the serotonin-selective reuptake inhibitor fluoxetine (10 mg/kg/i.p.). Twenty-four hr after the last injection the mice were sacrificed and hippocampal levels of p21 were analyzed by Western blot. All three antidepressants produced a marked decline in p21 protein levels in the hippocampus. We tested whether this effect is specific for p21. No differences were observed in the levels of the Cdk inhibitors p27 (Cip/Kip family) or p18 and p19 (Ink4/Arf family, data not shown for p19) (Fig. 5A,B).

BrdU, which incorporates into nuclear DNA of proliferating progenitors during the S phase of the cell cycle, was used to label proliferating cells. To confirm that the antidepressant-induced decreases in p21 were associated with increased neurogenesis, separate groups of mice were treated as described above. Twenty four hr after the last injection of drug (or saline), the mice were injected with BrdU and then sacrificed 24 hr later. The number of BrdU^+^ cells in the SGZ was significantly higher in antidepressant-treated compared to the saline-treated mice, showing that antidepressant treatment increased cellular proliferation (Fig. 5C). To determine the number of proliferating cells that expressed a neuronal phenotype, slides were double-labeled with both BrdU and DCX antibodies, and the number of BrdU^+^/DCX^+^ co-labeled cells (proliferating neuronal progenitors) were determined. The number of BrdU^+^/DCX^+^ cells was increased in all three antidepressant-treated groups compared to saline-treated controls (Fig. 5D). Therefore, chronic treatment with different classes of reuptake inhibitor-type antidepressants decreased hippocampal p21 expression and increased neurogenesis.

### p21-/- mice exhibit higher levels of neurogenesis in response to saline and lower levels in response to imipramine

**Figure 6:**
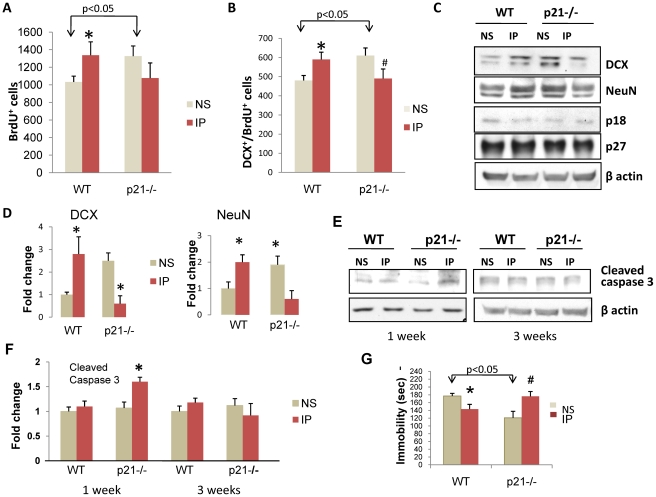
SGZ neurogenesis and immobility in the FST in p21-/- mice chronically treated with normal saline (NS) or imipramine (IP). **A**) Number of BrdU^+^ cells. The effects of treatment x genotype were assessed by two-way ANOVA followed by Neuman-Keuls post-hoc test [(F1,16,) = 20.18, p = 0.004]; **B**) Number of BrdU^+^/DCX^+^ cells in the SGZ. Two-way ANOVA [(F1,16) = 23.483, p = 0.00018]. Data are presented as a mean ± SEM, *, p<0.05 vs WT NS, #, p<0.05 vs p21-/- NS; Western blot analysis of **C**) Neuronal markers and Cdk inhibitors; and **D**) Cleaved caspase 3. For each sample hippocampi from 3 mice/group were pooled; **E, F**) Quantitative analysis of three independent experiments. The intensity of **E**) DCX and NeuN, and **F**) cleaved caspase 3 bands were measured, normalized to the loading control (β actin) and ratios were corrected to WT NS control to quantify relative changes. Data are presented as a mean ± SEM, *, p<0.05 vs WT NS; **G**) Immobility in the FST. The values are the total time spent immobile in sec. Data are presented as a mean ± SEM, n = 9–10/group *, p<0.05 vs WT NS or p21-/- NS, #, p<0.05 vs p21-/- NS. Imipramine decreased immobility in WT treated mice [(M = 143.08 SD = 39.02) compared to WT untreated (M = 176.99 SD = 20.39) mice, t(17) = 2.33, *P*  = 0.032]. The effects of treatment and genotype were assessed by two-way ANOVA followed by Neuman-Keuls post-hoc test[ F(1, 33) = 12.551, p = .001]. The p21-/- NS group had significantly less immobility time compared to WT NS whereas the p21-/- IP treated group had significantly more immobility than the p21-/- NS group. This indicates that antidepressant treatment produces a paradoxical effect in the p21-/- mice.

WT and p21-/- male mice were treated chronically with saline or imipramine as described above. Twenty four hr after the end of antidepressant treatment the mice were injected with BrdU as described above and BrdU^+^ cells were counted in the SGZ. Compared to WT mice that received saline, the number of BrdU^+^ and BrdU^+^/DCX^+^ co-labeled cells was significantly higher in the saline-treated p21-/- mice, indicating increased neurogenesis. Treatment with imipramine increased number of BrdU^+^ and BrdU^+^/DCX^+^ co-labeled cells in WT mice, but did not increase the number BrdU^+^ cells in p21-/- mice. There is a tendency for imipramine to decrease BrdU labeling in p21-/- mice, although this effect did not reach statistical significance. However, impramine significantly reduced the number of BrdU^+^/DCX^+^ cells in p21-/- mice (Fig. 6A and B). Thus, neurogenesis decreased rather than increased in p21-/- mice in response to treatment with imipramine. Western blot analyses of hippocampi from WT and p21-/- mice confirmed these observations (Fig. 6C). In WT mice, imipramine treatment increased protein levels of both DCX and NeuN (a marker of mature neurons), indicating increased neurogenesis. In the saline-treated controls, levels of both DCX and NeuN proteins were increased in p21-/- mice compared to WT mice, again showing increased neurogenesis in p21-/- mice. Although treatment with imipramine increased the level of these proteins in WT mice, the expression levels of both DCX and NeuN were markedly decreased after treatment with imipramine in p21-/- mice. No differences in the levels of two other major Cdk inhibitors p18 and p27 were found among treatment groups, indicating that the effects of the antidepressants in either WT or p21-/- mice do not involve these other Cdk inhibitors (Fig. 6C,D).

Caspase 3 is a member of the caspase family, and in apoptotic cell caspase is cleaved to shorter isoforms to trigger the apoptotic process [29]. After three weeks of imipramine treatment there were no differences in the levels of cleaved caspase 3 protein among treatment groups. However, after one week of imipramine treatment the levels of cleaved caspase 3 were higher in the p21-/- mice, indicating increased apoptosis (Fig. 6E,F). Apoptotic death of rapidly proliferating neuronal progenitors at an early stage (i.e., in 1 week) might explain the decrease in the number of BrdU^+^ and BrdU^+^/DCX^+^ cells and in DCX and NeuN protein levels observed later (i.e., in 3 weeks) in imipramine-treated p21-/- mice.

### p21-/- mice do not show decreased immobility in the FST in response to imipramine

For the FST, WT and p21-/- mice were treated chronically with saline or imipramine as described above. Twenty four hr after the end of antidepressant (or saline) treatment the mice underwent the FST. Compared to WT mice that received saline, there was less immobility in the saline-treated p21-/- mice (Fig. 6G). Although imipramine treatment decreased immobility in the WT mice, immobility was increased in the p21-/- mice. Thus, imipramine does not show antidepressant activity in the FST in p21-/- mice.

## Discussion

The data indicate that p21 is involved in the fundamental molecular mechanisms underlying the control of neurogenesis in the hippocampus. The present study confirmed previous findings that p21 is expressed exclusively in the SGZ of hippocampus, but not in granular cell layer where the mature neurons reside [15]. In the present study we found that p21 is expressed in transient amplifying progenitors, neuroblasts and young developing neurons. In NPC derived from the hippocampi of WT mice, p21 is co-localized with both SOX2 and nestin in early stage neuronal progenitors. After differentiation, GFAP^+^ astrocytes do not express p21, whereas p21 expression is evident in early stage both DCX^+^ and immature βIII-tubulin^+^ (Tuj-1^+^) neurons. Co-labeling for p21 and Ki67 show that these two proteins rarely co-localize. Therefore, when p21 is present, proliferation is restrained in these cells.

The *in vitro* studies support this hypothesis. Compared to neuropheres derived from WT mice, spheres derived from p21-/- mice were larger in size, the spheres were comprised of more cells and they exhibited higher spherogenic ability and increased proliferation. In addition, the percentage of both SOX2^+^ and nestin^+^ cells expressing Ki67 (proliferating cells) was higher in NPC derived from p21-/- mice. The experiments using conditional knock down of p21 in NPC from WT mice showed that when p21 expression is decreased, proliferation of NPC is increased. Taken together, these results strongly support the hypothesis that p21 plays a fundamental role in regulating neuronal lineage cell proliferation in the hippocampus and in keeping these cells in a quiescent state. *In vivo*, saline-treated p21-/- mice showed greater cellular proliferation in the SGZ (as detected by increased BrdU labeling) and increased neurogenesis (as indicated by more BrdU^+^/DCX^+^ co-labeled cells) compared to WT mice. These results are in agreement with the finding of increased proliferation in the lateral ventricle wall and in the forebrain stem cells in young p21-/- mice [30].

Other published studies [2], [6], [7] have shown that chronic treatment with nonselective, serotonin- and norepinephrine-selective reuptake inhibitor-type antidepressants stimulate hippocampal neurogenesis. In the present study this was found to be associated with decreased hippocampal p21 protein expression. No significant changes were observed in the expression of other Cdk inhibitors, showing that p21 plays an unique role in antidepressant-induced increases in neurogenesis. These results also indicate that a shared property of reuptake inhibitor-type antidepressant drugs is that they decrease the expression of p21 in the SGZ of the hippocampus. Although DMI appeared to have the greatest suppressive effect on p21 expression, there were no differences among the three antidepressants in terms of the proliferating neuronal progenitors (i.e., stimulation of neurogenesis). It is important to point out that it is not appropriate to try to draw conclusions regarding comparative potencies of the three drugs used because equimolar doses were not administered, nor were dose-response studies conducted.

Tissue specific factors can start or stop the proliferation of mitotic cells. Out of three tasted major Cdk inhibitors (p21, p18 and p27), it is only p21 that changed in response to antidepressant treatment. We can speculate that in the hippocampus, some tissue-specific factors selectively affect p21. Similar tissue specificity has been demonstrated for other brain regions. For example, TGFβ exerts its anti-proliferative properties in the cerebellum, but not in the hippocampus [31]. On the other hand, Smad7, one of the transcription factors of the TGFβ signaling pathway, induces proliferation of SGZ and SVZ neuronal progenitors, independent of TGFβ signaling [32]. We also examined other Cdk inhibitors using immunofluorescent analysis (not shown here) and found that p27 is most abundantly expressed in all parts of the dentate gyrus. Therefore, it is likely that p27 maintains quiescence of neuronal stem cells and differentiated neurons in the granular cell layer.

An important finding is that neurogenesis was not increased in p21-/- mice chronically treated with imipramine, suggesting that p21 suppression is not simply a correlate of increased neuronal cell proliferation, but that down-regulation of p21 is required for the stimulation of neurogenesis produced by this class of antidepressants. In fact, in p21-/- mice there was a decrease, rather than an increase in the number of both BrdU^+^ and DCX^+^/BrdU^+^ cells in response to chronic imipramine treatment. These observations were supported by the results showing that imipramine decreased protein levels of both DCX and NeuN in the p21-/- mice. The mechanism underlying the decrease is not clear. Cleaved caspase 3 is a protein implicated in cell death signaling pathways [33]. In other cell types, chronic treatment with antidepressants can induce apoptosis via caspase 3 activation [34], [35]. In the hippocampus, apoptosis occurs mostly in proliferating early neuronal progenitors [36], [37], however it might also occur, although at a slower rate, at the post-mitotic stage [37], [38]. In WT mice, chronic imipramine treatment did not induce changes in apoptosis as evident by cleaved caspase 3 levels. Besides its effects on cell cycle regulation, p21 also has antiapoptotic properties [39]. It is possible that in the absence of p21, NPC become vulnerable to antidepressant-induced apoptosis. Another possibility is that p21 might directly interact and inactivate caspase 3 and its downstream apoptotic pathway [40], and in the absence of p21, caspase 3 is activated and apoptosis is induced. This is supported by the finding that in p21-/- mice, cleaved caspase 3 protein levels were increased after one week of imipramine treatment, indicating induced apoptosis. This could explain the decreased levels of neurogenesis in p21-/- mice after three weeks of treatment with imipramine. The fact that no changes in the levels of cleaved caspase 3 were found after three weeks might indicate that by this time very few early neuronal progenitors proliferate in the SGZ of p21-/- mice.

Although there is evidence pointing to a mechanistic link between neurogenesis and the mechanism of action of antidepressant drugs, the role of neurogenesis in the pathophysiology of depression remains unclear. Suppression of neurogenesis by hippocampus-directed irradiation [10] or by using transgenic animals [41] does not produce a depressive or anxious phenotype per se, but does reduce some of the behavioral effects of antidepressants [10]. Recent findings also indicate that adult-born hippocampal neurons are required for normal stress response, and support a direct role of neurogenesis in stress-related depressive behavior [42]. The link between depression in human and increased immobility in the forced swim test in the mouse must be drawn with great caution [43], but in the present study there was a clear-cut inverse relationship between immobility in the FST and hippocampal neurogenesis. For example, saline-treated p21-/- mice showed less immobility in the FST and increased neurogenesis compared to saline-treated WT mice. However, treatment with imipramine reduced immobility and increased neurogenesis in the WT mice, but increased immobility and decreased neurogenesis in the p21-/- mice. The last imipramine injection was administered to the mice 24 hr before the FST, which rules out acute drug/genotype interactions. These results indicate that imipramine does not show antidepressant activity in the FST or stimulate neurogenesis in p21-/- mice, and strongly suggest that p21 is required for the stimulation of neurogenesis and some of the behavioral effects produced by reuptake inhibitor-type antidepressant drugs.

In summary, p21 regulates the proliferation of neuronal progenitors in the hippocampus, and the stimulation of neurogenesis produced by reuptake inhibitor-type antidepressants might be a consequence of decreased p21 expression and the subsequent release of neuronal progenitor cells from the blockade of proliferation. Because many antidepressants stimulate neurogenesis, it is possible that their shared common mechanism of action is suppression of p21. At this point the mechanisms underlying this effect are not known. As direct effects of the antidepressants on p21 expression in NPC *in vitro* were not found (data not shown), it is likely that antidepressants suppress p21 secondary to enhancing neurotransmission. Similar effects was shown for the long term lithium treatment which suppress p53 expression in cerebellum granular cells [44], and p21 is a transcriptional target for p53[45]. Further studies will be needed to determine the mechanisms and pathways underlying suppressive effects of antidepressants on p21 expression.
